# Extended Arm of Precision in Prosthodontics: Artificial Intelligence

**DOI:** 10.7759/cureus.30962

**Published:** 2022-11-01

**Authors:** Shriya R Singi, Seema Sathe, Amit R Reche, Akash Sibal, Namrata Mantri

**Affiliations:** 1 Department of Public Health Dentistry, Sharad Pawar Dental College and Hospital, Datta Meghe Institute of Medical Sciences (Deemed to be University), Wardha, IND; 2 Department of Prosthodontics, Sharad Pawar Dental College and Hospital, Datta Meghe Institute of Medical Sciences (Deemed to be University), Wardha, IND; 3 Department of Public Health Dentistry, Sharad Pawar Dental College and Hospital, Datta Meghe Institute of Medical Science (Deemed to be university), Wardha, IND

**Keywords:** maxillofacial prostheses, implantology, cad/cam, prosthodontics, artificial intelligence

## Abstract

Dentistry based on artificial intelligence (AI) is not a myth but turning into a reality. AI has revolutionized medicine and dentistry in various ways. AI is a technology that uses machines to imitate intelligent human behavior. AI is gaining popularity worldwide because of its significant impact and breakthrough in the field of intelligence innovation. It is a lifesaver in dentistry, particularly in the field of prosthodontics, because it aids in the design of prostheses and the fabrication of functional maxillofacial appliances. It also helps in the processes of patient documentation, diagnosis, treatment planning, and patient management, allowing oral healthcare professionals to work smarter rather than harder. While it cannot replace the work of a dentist because dentistry is not about disease diagnosis, it does involve correlation with other clinical findings and provides treatment to the patient. The integration of AI and digitization has brought a new paradigm in dentistry, with extremely promising prospects. The availability of insufficient and inaccurate data is now the only barrier to the deployment of AI. Therefore, dentists and clinicians must focus on collecting and entering authentic data into their database, which will be completely utilized for AI in dentistry shortly. This study focuses on various applications of AI in prosthodontics along with its limitations and future scope.

## Introduction and background

As we all know, the world is rapidly moving toward digitalization in many aspects of life. This digital technology has made living even easier. This digital revolution has transformed dentistry in every way and enhanced the quality of care [1]. For as long as history has been recorded, scholars and technologists have been fascinated by the functioning of the brain. And while various technologies have evolved based on ideas that attempt to replicate the functioning of the human brain, a machine that can think like a human is still a dream [2]. Over the years, man has worked to create technologies that can accurately imitate the working of the human brain, which has resulted in the development of a technology known as artificial intelligence (AI). AI is described as “a branch of science and engineering concerned with the computational understanding of what is often referred to as intelligent behavior and the development of artifacts that display such behaviour” [3]. Expert systems, theorem-proving, game playing, picture recognition, natural language processing, and robotics are all examples of uses of AI in telecommunications and aerospace. In the recent decade, technology has also changed the fields of medicine and dentistry [4]. AI advancement can be a boon to dental healthcare workers by reducing stressful work and manpower.

**Figure 1:**
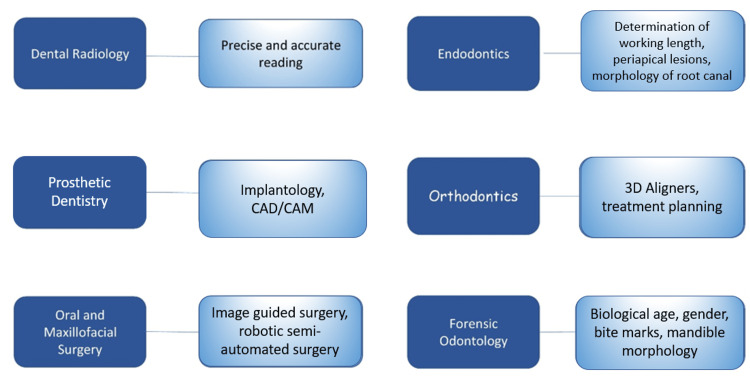
Applications of AI in dentistry AI, artificial intelligence; CAD/CAM, computer-aided design/computer-aided manufacturing Open access journal under a CC-BY license Contributed by Agrawal P, Artificial Intelligence in Dentistry: Past, Present, and Future [6].

There is a vast amount of data in healthcare systems that provide optimal learning sources for machine learning-enabled decision support systems. This support system can help healthcare practitioners sort out the intricacies of clinical variabilities and increase diagnostic accuracy. With the development of cloud computing capability, data processing, and the availability of massive amounts of data collected, AI in general, and AI in healthcare or dentistry in particular, began to gain momentum. A specific algorithm was established with the vast amount of data available, for example, in the field of radiology, which further aided in diagnosing and providing probable treatment alternatives [5]. It has the potential to be used in prosthodontics, orthodontics, oral surgery, and periodontics for condition analysis and treatment planning. Various applications of AI in dentistry are shown in Figure 1.

## Review

AI in prosthodontics

**Figure 2:**
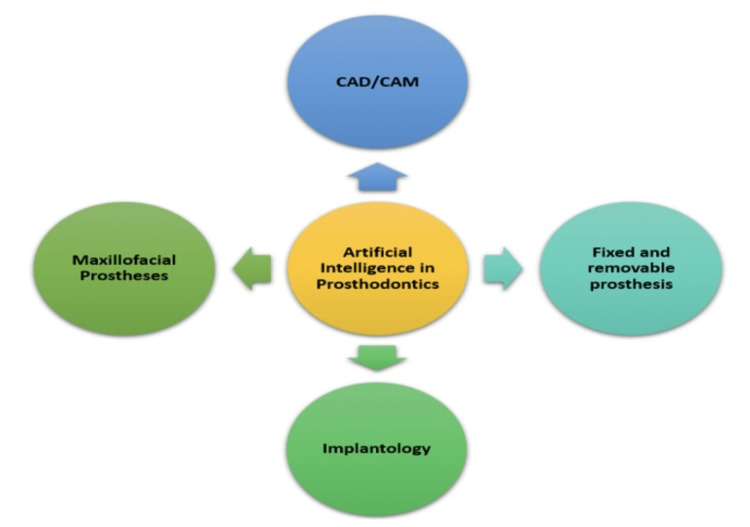
Applications of AI in prosthodontics. AI, artificial intelligence; CAD/CAM, computer-aided design/computer-aided manufacturing Figure credits: Shriya Singi

Prosthodontics is the *art and science of dentistry that deals with the diagnosis, treatment planning, rehabilitation, and preservation of the oral structures' function, comfort, aesthetics, and health of patients with clinical problems associated with missing or deficient teeth and oral and maxillofacial tissues*. It deals with this largely through prosthetic replacement [7]. Prosthodontics is primarily concerned with the treatment and fabrication of removable and fixed dental prostheses, as well as the preparation of finishing margins alongside the tooth for better extension and fitting of the prosthesis, implant surgery, and the construction of a maxillofacial prosthesis. It is also used for maintaining maxillomandibular relations and tooth shade selection for improved appearance [8]. AI can be quite beneficial in a variety of therapy protocols. This paper discusses several applications of AI in prosthodontics which are also summarized in Figure 2.

Computer-aided design/computer-aided manufacturing

In prosthetic dentistry, computer-aided design/computer-aided manufacturing (CAD/CAM) is gaining popularity [9]. AI integration with CAD/CAM increases its chairside use [10]. To save time and energy, the computer has a designing and manufacturing unit that allows us to design mill or print based on the patient's preferences. The CAD/CAM system typically builds two- and three-dimensional models, which are then materialized using numerically controlled mechanics [11].

The CAD/CAM approach aids in the scanning of prepared teeth and the fabrication of restorations with milling using ceramic blocks. Because no casting procedures are necessary, manufacturing prosthetic restoration has become easy [12]. Inlays, Onlays, and crowns as well as crowns and bridges are manufactured using CAD/CAM technology [13]. It has replaced the laborious and time-consuming process of traditional casting while also decreasing the human error component in the final prosthesis [14]. Dentists are utilizing AI to assist them in designing the best possible and aesthetically pleasing prosthesis for patients while taking a range of parameters into account, such as facial dimensions, ethnicity, anthropological calculations, and even the patient's demand [15,16]. The prosthesis is currently made using CAD/CAM technologies such as additive manufacturing and subtractive milling technologies such as 3D printing.

It assists with the design of the restoration and mills a restoration with great precision to provide improved function and aesthetics. Integrating AI with CAD/CAM technology into removable denture design and manufacture has improved denture quality while also simplifying laboratory operations. Manual laboratory procedures are decreased or eliminated, allowing the dental technician and the dentist to ensure prosthesis reproducibility and accuracy. This minimizes the overall time needed for patient rehabilitation [17].

Tooth-supported fixed and removable prosthesis

Removable partial dentures (RPDs) supported by teeth or implants are a less invasive and cost-effective treatment option for restoring missing teeth [18-20]. Designing the various aspects of an RPD is an important step in the fabrication of a prosthesis [21,22]. Similarly, AI algorithms have been developed to assist in the design of RPDs. In the case of fixed dental prostheses, the original tooth structure will be scanned and software will be utilized to evaluate and propose alternative treatment options. For partial edentulism, the application will utilize software to recommend an RPD design [23].

In removable prosthodontics, a clinical decision support model that applies case-based reasoning and ontology proved capable of proposing the design of individualized RPDs. However, this model bases its recommendations on the most likely example in the database. Because clinical settings are constantly varying, it is necessary to keep a dubious approach to its output [23]. An artificially intelligent system in fixed prosthodontics is still under development. The key feature of AI is its ability to analyze and learn from the millions of doctor-approved crowns in the database, which is constantly being added to the cloud regularly. The computer evaluates how each high-performance restoration is built to achieve optimal function, depending on the perfect occlusion, contacts, and margins suited for each case, to learn from successful crown designs [16].

Originally, tooth margin preparations during the production of the fixed dental prosthesis were done manually by dentists using a handpiece and a variety of burs. Optimal contour and extension of the marginal line surrounding the teeth assisted in keeping the prosthesis in place while also providing a healthy environment and protection to the gums and periodontium. These methods necessitated more advanced technical abilities as well as more time. The goal was to eliminate the time-consuming manual labor and errors. Zhang et al. [24] conducted deep learning (DL) model research to extract marginal lines with precision. There were 380 dental preparation models in this study. To extract the data, a Convolutional Neural Network (CNN) model, called Sparse Octree (S-Octree), was utilized. With the assistance of the dental preparation procedure, a sparse point cloud with labels was created. For the study, an eight-depth octree structure was created. The data was divided into three sets: training, verification, and testing. CNN models were built by labeling dental preparations. Back-projection and boundary extraction methods were used in the study, and a tooth preparation line was extracted to address the drawbacks of manual practice. The average precision reached was 97.43%. This increased accuracy demonstrated AI's ability to overcome manual errors, making it a viable alternative for adoption [24].

Implantology

Treatment plans for dental implants can be successful when the cone-beam CT (CBCT) image and intraoral scans are combined. The application of AI in implantology offers the potential to combine both and develop future prosthetics [23]. To consistently and automatically figure out the precise location of the mandibular canal for dental implant surgeries, experts from the Finnish Centre for Artificial Intelligence; the Alan Turing Institute, Planmeca; and the University Hospital of Tampere suggested a new model. Applying DL-based object recognition, implant systems can be detected from panoramic radiographic scans [25]. AI has been implemented in implant dentistry to detect implant types from periapical and panoramic radiographs [26-28].

With the introduction of implantology, numerous constraints on fixed and removable prostheses can be resolved. Implants have the benefit of being more resistant to dental disorders, providing stronger support, and preserving residual ridge in cases of distal extension [29]. Implants are extensively used in dentistry to replace lost teeth or to restore the entire mouth. Because of improved aesthetics and stability, implant prostheses have gained popularity in recent years. There are approximately 4,000 dental implants sold worldwide. They differ in terms of treatment procedures and structures. A dentist must appropriately identify and classify implants to avoid replantation and repair owing to biological and mechanical complications. The basic approaches for classifying implant structures are CAD/CAM and panoramic radiography [8]. Digital technology allows for the digital planning and placement of extraoral implants, as well as the design and fabrication of maxillofacial prostheses. A significant benefit of digital planning is that clinicians can envision the ideal implant placements and positions on a computer screen before surgery, after which a surgical guide can be virtually created and built using rapid prototyping (RP) technology [30].

When standard CAD/CAM technologies are applied for implant prosthesis cementation, various problems can occur. Positional mistakes, cementation errors, and occlusal or interproximal correction with an abutment can all cause errors [8]. Lerner et al. [31] presented an AI model to reduce these errors. This AI model was designed to aid in the creation of fixed implant prostheses with monolithic zirconia crowns. The use of an AI model to aid in the detection of abutment subgingival margins. This model also assisted the dentist in focusing on tooth preparation and maintaining interproximal and occlusal contacts. This convenience was intended to eliminate errors and time consumption. Patient data from 2016 to 2019 was used in the study using zirconia implant prostheses in the posterior teeth. The study's gender diversity was 7:11 in 90 patients, with a male-to-female ratio of 7:11. This study included a total of 106 implants. Intraoral scans, radiographs, pictures, and CAD scenes were among the data sets used to develop AI models (images). The use of an AI model in the production of zirconia implants for the posterior teeth yielded encouraging results, with a survival rate of 91% and a success rate of 93%. Because the AI model's results revealed a high survival and success rate, it demonstrated the model's ability to be integrated into this field [31].

Before the actual surgery, AI software has assisted in the planning of surgeries down to the finest detail. The use of research-based, clinically established methodologies and technologies will standardize dental implant therapy. Following the acquisition of an intraoral and CBCT scan, based on the tissue thickness, bone type/thickness, emergence profile, and the patient's individual medical history, AI will automatically combine the two scans, design the future restoration, and then insert the suitable implant with the proper design in the optimal position. After that, the surgical guide can be created, and the procedure can be performed [23].

Predictive AI models can help in two areas of dental implantology. First, Machine Learning algorithms were used to build predictive model designs concentrating on bone levels and individual clinical outcomes. By simultaneously evaluating the implant system, patients' data, and surgeons' operations, a recurrent artificial neural network (ANN) with memetic search optimization produced 99.2% efficiency in success rate predictions [32]. Second, AI was offered as a replacement for present technology in anticipating the mechanical properties of a bioimplant system, reducing the high cost of computation involved with improving implant design variables. The application of AI in the risk optimization of bioimplants requires further development [24].

Maxillofacial prostheses

Maxillofacial prosthesis rehabilitation restores function and aesthetics due to facial abnormalities or injuries by replacing missing structures. Patients develop maxillofacial deformities as a result of trauma, cancer, or congenital illnesses. Because of the associated aesthetic and psychological issues, such defects frequently necessitate high-quality prosthetic repair [30]. It can be difficult to produce excellent aesthetic outcomes when reconstructing maxillofacial abnormalities in many circumstances. Maxillofacial prosthodontists offer a variety of alternatives for rehabilitating patients with prosthetic restorations to improve function and aesthetics. Without risks associated with surgery, an aesthetic and functional maxillofacial prosthesis ameliorates patients' anxieties and improves their quality of life. Digital technology allows for the digital planning and placement of extraoral implants, as well as the design and fabrication of maxillofacial prostheses. Before CAD/CAM technology, reconstructing a facial form with a maxillofacial prosthesis required skilled hand carving of a wax cast. Computer developments have enabled the digital design of maxillofacial prostheses [14,33]. When using CAD/CAM technology to fabricate maxillofacial prostheses, a common treatment process begins with imaging techniques that capture the patient's soft and hard structures (eg, MRI and CT). This data is then translated into an RP model using computer software (eg, Materialise Mimics, Leuven, Belgium). RP models can be printed in wax directly or in acrylic resin and then translated into a wax cast using replication procedures. Because RP techniques cannot precisely simulate skin curvature, the wax cast is fitted to the patient and final minute features must be hand carved. Following fitting on the cast, the silicone elastomer prostheses are routinely constructed [34-36]. When the natural facial structure (e.g., the nose) is deformed, CAD/CAM technology can be utilized to create immediate maxillofacial prosthetics with a form chosen from a digital library. This method takes less time than the traditional method.

The bionic eye, developed in the United States, has already been trialed on a dozen people who have lost their vision. These technologies, which use AI, can help patients achieve vision without requiring surgery. In this approach, a smart camera mounted on special glasses enables the user to understand the text or recognize faces. An expert analyses the camera's data and converts it into audio, which is then given to the visually impaired person's ears via a wireless earpiece. Patients who have had limbs amputated may lose sensory capability in those locations. This scenario is changing because of artificial skin developed by researchers at the Federal Polytechnic School of Zurich, Zurich, Switzerland, and the California Institute of Technology, Pasadena, CA, USA. The tissue, which is made up of a thin, translucent coating of water and pectin, detects temperature fluctuations between 5 and 50 °C [25]. Artificial olfaction is essential in robotics because it simulates the human olfactory structure, which can recognize unique smells in a wide range of sectors such as disease diagnosis, environmental monitoring, public security issues, the food industry, and agricultural production [37].

Limitations

AI can be utilized to do tasks in record time. It can aid in the standardization of many procedures. AI aids in the making of logical and realistic conclusions, resulting in an accurate diagnosis. Although there are certain drawbacks to applying AI in dentistry, such as system complexity, adequate training, and costly setup. Data is usually used for both training and testing, which leads to *data snooping bias*. The findings of AI in dentistry are not immediately applicable [38].

**Figure 3:**
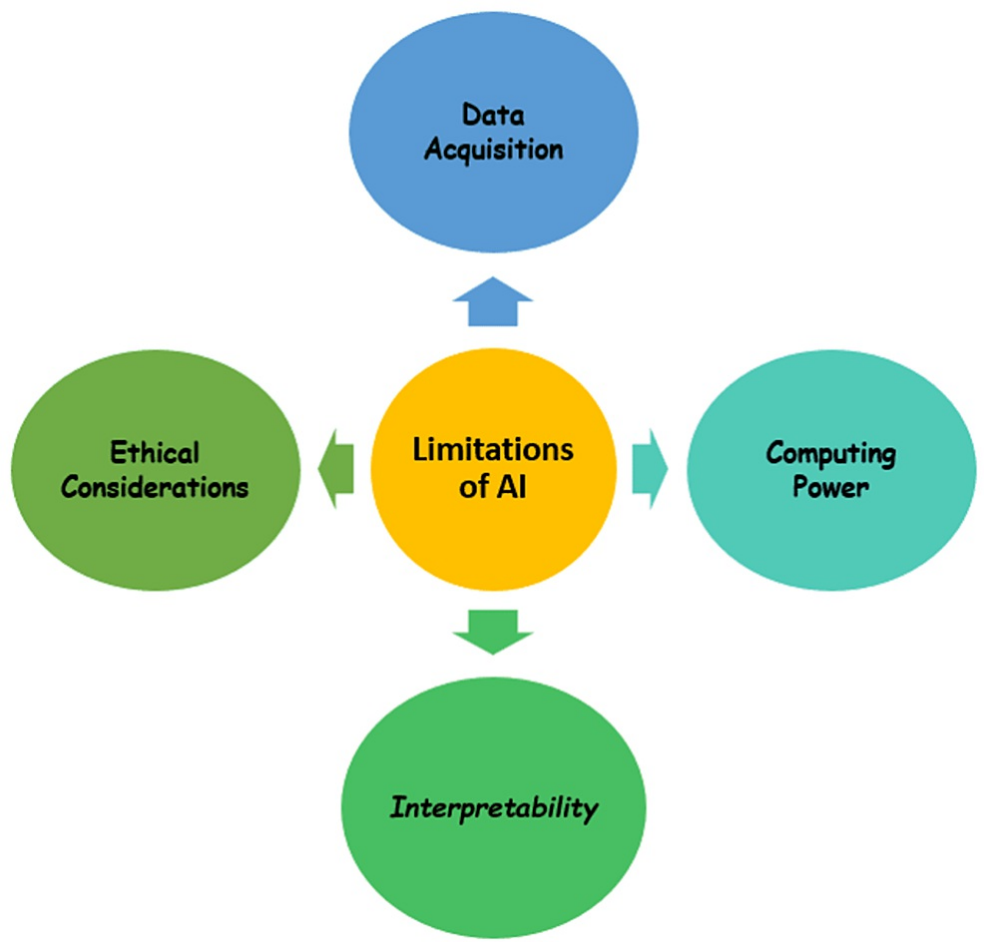
Limitations of artificial intelligence. Figure credits: Shriya Singi

Using AI to address problems necessitates a comprehensive algorithm with several applications to respond to a given query. AI will not provide direct interpretation; misinterpretation may arise due to algorithm malfeasance. To reduce the possible hazards of AI, AI programs must still be built in collaboration with experienced physicians and professional computer engineers. Another developing risk will be a liability if the diagnostic job becomes overdependent on the AI system [39]. When analyzing information offered by AI, clinicians should constantly be vigilant and careful. For the most part, machine learning requires data for training. To prevent breaking the Health Insurance Portability and Accountability Act of 1996 (HIPAA) laws, the exchange of training sets and application of models should be done with prudence [23]. The main drawbacks of using AI are highlighted in Figure 3.

Data Acquisition

AI relied significantly on data sets. For optimal model training, these data sets should be adequately categorized and filtered. The limitations were that most data were in paper format and data consolidation was not done appropriately due to a lack of awareness in follow-up care. In the current situation, the medical sector has commenced digitization in the diagnoses and reports, but there is still a long way to go for reliable statistics that can be used in training the model [8]. Dentistry is several years behind medicine in terms of AI application. The sample size for testing and training in addition to the information for standard and comparative tests, is occasionally ambiguous, raising doubts about the comparability, robustness, and generalizability of the results [40]. It is critical to standardize methodology in data collection and reporting to increase data quantity, quality, and readability. Creating a standard open-access data set, including complete clinical, experimental, and therapeutic data, would be a critical job in the next stage of AI research to allow for the analysis and comparison of various algorithms.

Computing Power

Collecting information from constantly updated healthcare databases for AI applications necessitates continuous processing power upgrades. Because the computational power of traditional computers has been fully exhausted, a lack of computational resources in data processing has become one of the barriers to AI's productivity [41]. Quantum computing uses quantum bits (qubits) to process data in line with the laws and constraints of quantum mechanics. The capacity of quantum computing to manage data from highly varied data sets at the same time makes it a desirable platform for accelerating AI, particularly machine learning, spanning computer software to data collecting and modeling.

Interpretability

Interpretability is important for two reasons. First, it is critical for the relationship between humans and technology to ensure that software is a realistic interpretation of medical situations. Failure to elucidate AI's fundamental workings will inevitably undermine practitioners' confidence in its clinical significance. Second, the lack of interpretability and transparency makes predicting failures and generalizing specific techniques for similar scenarios problematic. Improving visualization has become a critical endeavor. Thorough scientific research and treatment approaches that react to patient and practitioner narrative interactions are necessary to comprehend the clinical phenotypes of AI and their relevance to customized care [42].

Ethical Considerations

AI development should assure that these techniques do not harm individuals while also maintaining the moral status of the machines themselves [43]. These ethical conundrums highlight the importance of establishing explicit criteria for the clinical application of AI. Another ethical problem is the determination of legal liability. AI is currently unaccountable, whether it is deployed supervised or unsupervised. The dentist is accountable for each patient and how information is utilized. Applying the same ethical and social norms that humans accept is unacceptable when the line between humans and machine accountability is becoming increasingly blurred with the introduction of chatbot-based, unsupervised AI-based diagnoses [44].

Future scope

The future of AI implementation was intriguing as it enabled decentralization in the treatment process. AI has improved remote therapy for medical practitioners. The accuracy of disease diagnosis will improve in the future as AI makes predictions that may be combined with human diagnosis to improve the chances of correct diagnosis [8]. AI will certainly continue to link with a dental practice in all aspects due to the necessity for precision and quick information sharing in dentistry. Dental practitioners will benefit from technologies that would let them make accurate diagnoses and recommendations for complete treatment plans, as well as calculate the alternatives for each of these in a short period. The involvement of AI in treatment planning will enable various multidisciplinary treatment ideas, with advantages and potential complications based on acquired evidence. This proof base’s information from a scientific database will be updated in real-time. However, doctors who receive “AI guidance” options will be responsible for selecting the most appropriate option on their own [23].

With the latest advancement in CAD/CAM technology, and materials demanding a superior level of precision in prosthodontics, laboratories designing algorithms with AI capabilities will be in huge demand. This program will assist laboratory technicians in designing prostheses with ideal aesthetics, hygienic contours, and low failure rates. For fixed prostheses, an optical intraoral scanner will be used to scan the present tooth structure and software will be used to analyze and propose therapeutic choices. In the case of partial edentulism, the program will be able to use software to suggest the design of RPDs. The use of research-based, clinically established technologies and methods will standardize dental implant therapy.

As more AI-based services join the market, the benefits to the dentist's practice become evident. Big data enables a variety of approaches to using AI to make business choices in practice. Nonetheless, it is important, to note that dental practitioners must have a fundamental knowledge of how AI software is designed and data is collected. Knowing the benefits and present limits of AI technology will help clinicians choose the right AI service as additional services join the market.

The usage of AI in dental insurance will progress and, eventually, enable rapid claim approvals. This will enable doctors to upload intraoral scans, radiographs, and pictures to an insurance provider and receive a quick response to their insurance claim, enabling accountability in the process and permitting patients to receive better dental care without concern of not having insurance coverage. AI will improve the dental patient experience in addition to clinical approaches. The system will understand patient preferences to provide a better overall experience. By improving the dental patient experience, more patients will receive optimal oral health treatment, resulting in better systemic health [23].

## Conclusions

In the recent decade, the domain of AI has evolved enormously. The most exciting AI applications are on the horizon, and they are about to enhance the domain of dentistry. AI is rapidly evolving, but it will never be able to replace human knowledge, talent, and decision-making ability. The application of AI in prosthodontics is expanding at an exponential rate. The outcomes of the implementation are comparable to, and occasionally better than, those of humans. AI can be viewed as a possible tool in every aspect, such as classifying denture fixtures and maxillofacial prostheses, extracting marginal lines, and reducing human error in implant cementation. Furthermore, AI can only assist clinicians in performing jobs professionally; it cannot replace human knowledge, ability, or treatment planning. Although challenges such as data collection, interpretation, computer power, and ethical difficulties exist and must be overcome, AI is widely regarded as an excellent auxiliary for dentists. AI can be unbiased, reproducible, user-friendly, and transparent with careful design and long-term clinical validation. Future AI development should continue to prioritize human interests while also improving its ability to handle large amounts of data. Although AI can help in a variety of ways, the final decision must be made by a dentist because dentistry is a multidisciplinary approach. Researchers are using AI to improve oral health and hygiene. Researchers are using AI to improve oral health as well as overall health.
